# Characteristics of Tau Oligomers

**DOI:** 10.3389/fneur.2013.00102

**Published:** 2013-07-19

**Authors:** Yan Ren, Naruhiko Sahara

**Affiliations:** ^1^Department of Neuroscience, Center for Translational Research in Neurodegenerative Disease, College of Medicine, University of Florida, Gainesville, FL, USA

**Keywords:** tau, oligomers, dimer, sarkosyl-insoluble, antibody

## Abstract

In Alzheimer disease (AD) and other tauopathies, microtubule-associated protein tau becomes hyperphosphorylated, undergoes conformational changes, aggregates, eventually becoming neurofibrillary tangles (NFTs). As accumulating evidence suggests that NFTs themselves may not be toxic, attention is now turning toward the role of intermediate tau oligomers in AD pathophysiology. Sarkosyl extraction is a standard protocol for investigating insoluble tau aggregates in brains. There is a growing consensus that sarkosyl-insoluble tau correlates with the pathological features of tauopathy. While sarkosyl-insoluble tau from tauopathy brains has been well characterized as a pool of filamentous tau, other dimers, multimers, and granules of tau are much less well understood. There are protocols for identifying these tau oligomers. In this mini review, we discuss the characteristics of tau oligomers isolated via different methods and materials.

## Introduction

Tau is a phospho-protein that belongs to the family of microtubule (MT)-associated proteins. The primary function of tau protein is to modulate MT dynamics for maintaining neuronal processes and regulating axonal transport. During pathogenesis, tau protein abnormally aggregates into intracellular, filamentous inclusions, or neurofibrillary tangles (NFTs) in the brains of individuals with neurodegenerative disorders. These are termed tauopathies [reviewed in Ref. (1)].

In human tauopathies, intracellular aggregates of abnormally hyperphosphorylated tau protein and neuronal cell loss typically coincide within the same brain regions (2). Several transgenic mouse models that overexpress human tau protein have demonstrated how tau pathology and neuronal loss progresses [mouse models are reviewed in Ref. (3)]. However, recent data suggest that tau is involved in neuronal dysfunction before NFTs are formed (4, 5). *In vitro* tau polymerization studies indicated that NFT formation consists of several steps: dimerization, multimerization, oligomerization, and protofibril formation (6–11).

About two decades ago, tau aggregation intermediates (also referred to as AD P-tau) were isolated from the buffer-soluble fraction derived from brains of AD patients (12). More than 10 years later, attention has focused on oligomeric tau species in human (13) and transgenic mouse (14) brains in order to identify the exact neurotoxic components of tau protein. However, the potential role of tau oligomers is poorly understood because they exist in various states (e.g., dimers, multimers, and granules). Here, we review various protocols used to isolate tau oligomers and propose a general outline for the identification of tau oligomers.

## Soluble Pre-Fibrillar Tau in Human AD Brains

Greenberg and Davies first reported to isolate sarkosyl-insoluble tau from paired helical filament (PHF)-enriched fraction from human AD brain homogenates (15). Cortical gray matter was homogenized in buffer containing 10 mM Tris-HCl (pH 7.4), 1 mM EGTA, 0.8 M NaCl, and 10% sucrose, and then centrifuged at 27,200 × *g*. PHF-associated tau was enriched from the supernatant by taking advantage of their insolubility in the presence of a detergent, sarkosyl. PHF-associated tau migrated at around 57–68 kDa on one-dimensional PAGE gels. This enriched supernatant was more acidic on two-dimensional PAGE gels compared to extracts from normal brains. Although this PHF-associated tau was not extracted from highly insoluble fraction containing NFTs, the sarkosyl-insoluble tau displayed the same structural and antigenic properties as PHFs isolated from NFTs (16–18) and was distinguishable from normal, soluble tau proteins.

Kopke et al. isolated non-PHF hyperphosphorylated tau from AD brains (12). In their protocol, cortical gray matter was homogenized in buffer containing 20 mM Tris-HCl (pH 8.0), 0.32 M sucrose, 10 mM β-mercaptoethanol, 5 mM EGTA, 1 mM EDTA, 5 mM MgSO_4_, and proteinase inhibitors. The homogenate was then subjected to differential centrifugation, and the fraction resulting from centrifugation between 27,000 and 200,000 × *g* was collected. This fraction was further extracted with 8 M urea to separate out the PHF-enriched pool. The supernatant contained abnormally phosphorylated non-PHF tau. These tau species were named AD P-tau and had a molecular weight of 67–70 kDa. They were three to fourfold more phosphorylated than tau extracted from control brains and could be detected by Tau1 antibody after alkaline phosphatase treatment. These highly phosphorylated AD P-tau proteins lost their normal MT assembly-promoting activity, which could be recovered upon dephosphorylation with alkaline phosphatase (19). Moreover, AD P-tau could sequester normal tau into filamentous tau aggregates, resulting in MT de-polymerization (20).

These studies suggest that a pool of intermediate pathological tau species exists and that this pool can be recovered in buffer-soluble fractions. The physiological activity and function of these tau species is reduced compared to normal tau species due to hyperphosphorylation. Since the intracellular mobility dynamics of these intermediate tau species is much greater than that of condensed tau aggregates in NFTs, it is possible that intermediate tau species induce neuronal death and/or synaptic dysfunction. Therefore, the isolation and characterization of these tau species is paramount for understanding the pathogenesis of AD and for searching therapeutic methods.

## Tau Oligomers in Mouse Models of Tauopathy

The first tau transgenic mouse model of frontotemporal dementia with Parkinsonism linked to tau on chromosome 17 (FTDP-17-Tau) was the JNPL3 line, which overexpresses P301L mutant 4R0N tau (21). The biochemical characterization of insoluble tau in these mice was done by a modified Greenberg and Davies method (21, 22). In this protocol, mouse brains were homogenized in buffer containing 25 mM Tris-HCl (pH 7.4), 150 mM NaCl, 1 mM EDTA, 1 mM EGTA, phosphatase inhibitors, and protease inhibitors. A pellet collected from 150,000 × *g* centrifugation was re-homogenized in high-salt/sucrose buffer [10 mM Tris-HCl (pH 7.4), 0.8 M NaCl, 10% sucrose, 1 mM EGTA, 1 mM PMSF] and centrifuged again at 150, 000 × *g*. The resulting supernatant was incubated with 1% sarkosyl, and then centrifuged at 150, 000 × *g*. The pellet was re-suspended in TBS as the sarkosyl-insoluble fraction. A 64-kDa tau predominantly existed in the sarkosyl-insoluble fraction; this tau was phosphorylated at multiple sites. Most notably, the amount of 64 kDa tau increased in an age-dependent manner, correlating well with the pathogenesis in JNPL3 mouse brain.

Noble et al. proposed a slightly different protocol for tissue homogenization using RIPA buffer without SDS (23). This modification allows for the study of both cytosolic and membrane-associated proteins involved in AD pathogenesis, such as amyloid precursor protein (APP), in the same extracts (24).

Another broadly used tauopathy mouse model is rTg4510 mice, which express repressible P301L mutant 4R0N tau and develop progressive age-related NFTs, neuronal loss, and behavioral impairment (5). Using a protocol similar to the one for JNPL3 mice with an additional 13,000 × *g* centrifugation as the first step, Berger et al. identified 140 and 170 kDa multimeric tau species in rTg4510 mouse brains (14). The 140 kDa tau was recovered in the supernatant fraction resulting from 150,000 × *g* centrifugation, while the 170 kDa tau was mostly in the sarkosyl-insoluble fraction. Both multimers were not affected by the presence or absence of reducing agent, indicating that the multimers are disulfide-bond independent. Importantly, the accumulation of 140 kDa tau coincided with the behavioral impairments of rTg4510 mice (14). Although this finding has had a huge impact on our understanding the neurotoxic mechanisms of tau oligomers, it is still unclear whether 140 and 170 kDa tau multimers can induce neuronal dysfunction. This is because these multimers comprise such a small proportion of the total tau pool (roughly < 0.1% of total tau, as estimated by Western blot signal). It should be noted that tau multimers with apparent molecular weights of ∼140 and ∼170 kDa are in fact tau dimers of ∼120 and ∼130 kDa, based on Bis-Tris or Tris-acetate SDS-PAGE migration (11, 25). This was further supported by mass spectrometry analysis of cross-linked tau dimers (26).

Most recently, we demonstrated that TBS-extractable 64 kDa tau species represents better the species involved in the progression of brain atrophy than does the sarkosyl-insoluble tau species (25). These 64 kDa tau species can be recovered in the supernatant following centrifugation of brain homogenates at 27,000 × *g* and further separation from normal tau by 150,000 × *g* centrifugation. TBS-extractable 64 kDa tau and normal tau are similar in thermo-stability but differ in other properties. Under non-reducing gel electrophoresis conditions, nearly all 64 kDa tau species are detected as dimers (∼130 kDa, according to size of molecular markers), whereas most normal tau proteins are detected as monomers. Immuno-electron microscopy revealed that the TBS-extractable 64 kDa tau enriched fraction contains tau-positive granules and filaments (25). This morphological finding was supported by MC1 immunoreactivity and Ab39 insensitivity (25). The MC1 antibody recognizes an early pathogenic conformation of tau (27), while the Ab39 antibody only detects mature tangles (28, 29). Overall, the characteristics of TBS-extractable 64 kDa tau are similar to AD P-tau from human brains.

## *In vitro* Tau Oligomerization

With tau assembly modeled *in vitro*, unphosphorylated recombinant tau can be polymerized by inducers such as heparin, heparan sulfate, polyunsaturated fatty acids, RNA, or quinones (30–34). Using the heparin-induced tau self-assembly method, we produced and isolated granular-shaped tau oligomers from soluble tau and filamentous tau by sucrose density gradient ultracentrifugation (10). These granular tau oligomers were morphologically defined by atomic force microscopy (AFM) to be 15–25 nm granules, and their molecular mass corresponded to about 40 tau molecules (10). Once formed, granular tau can continue to form filaments without any inducers in a concentration-dependent manner (10).

More recently, Lasagna-Reeves et al. proposed a method to prepare tau oligomers by using amyloid seeds (35). In their protocol, tau oligomerization can be induced in a relatively short period (1 h incubation at room temperature) after adding Aβ42 oligomers. After a total of three rounds of seeding procedures, Aβ42 seeds could be diluted to below the detection limit (35). Examination of these tau oligomers by transmission electron microscopy or AFM revealed a spherical morphology (35). This unique method of producing tau oligomers is a reasonable representative model supporting the amyloid hypothesis (36, 37), in which Aβ oligomers trigger NFT formation. Interestingly, tau oligomers, but not tau monomers or tau fibrils, can cause memory impairment in wild-type mice (13) and can decrease long-term potentiation in hippocampal brain slices (38).

The production of granular tau oligomers must be initiated by dimerization of tau monomers. Heparin-induced tau polymerization allows us to detect initial dimers because of the relatively slower kinetics compared to arachidonic acid-induced tau polymerization (6, 39). It begins by increasing the formation of cysteine-dependent dimers, which occur prior to the detection of thioflavin T (ThT) binding (11). The kinetics of tau polymerization is dependent on oxidative/reducing state. Higher-order oligomers and aggregates assemble more rapidly in the absence of the reducing agent dithiothreitol (DTT) (11). However, cysless-tau (4R tau with both C291A and C322A mutations) forms dimers, which eventually aggregate into fibrils after 24 h incubation with heparin, suggesting that tau aggregation occurs without disulfide-bond formation (11).

Two distinct tau dimers (i.e., cysteine-dependent and cysteine-independent dimers) have been identified in tauopathy mouse models, including JNPL3 mice (11) and rTg4510 mice (14, 25). These dimers have also been shown in cell cultures (11, 40). Dimer formation is an essential step for their further assembly into higher-order oligomers. Although these dimers themselves may not exist in a steady state, it is important to detect the initial step of tau dimerization.

## Generation of Tau Oligomer-Specific Antibodies

A monoclonal antibody that selectively recognizes tau dimers and higher-order oligomers has been generated by Binder’s group (26). This antibody, named tau oligomeric complex 1 (TOC1), was made against benzophenone-4-maleimide cross-linked recombinant tau dimers (26). Immunogold labeling and dot-blot analysis of aggregated recombinant tau revealed that TOC1 selectively labels tau dimers or oligomers but not filaments (26). Based on their preliminary mapping of the TOC1 epitope, the proline-rich region (Gly155-Gln244) and the C-terminal portion (Leu376-Ser421) were identified as potential binding segments for forming cross-linked tau dimer (26). Since these two regions are on the opposite sides of the MT-binding domains, Patterson et al. advanced the idea of the formation of an antiparallel dimer (26).

**Figure 1:**
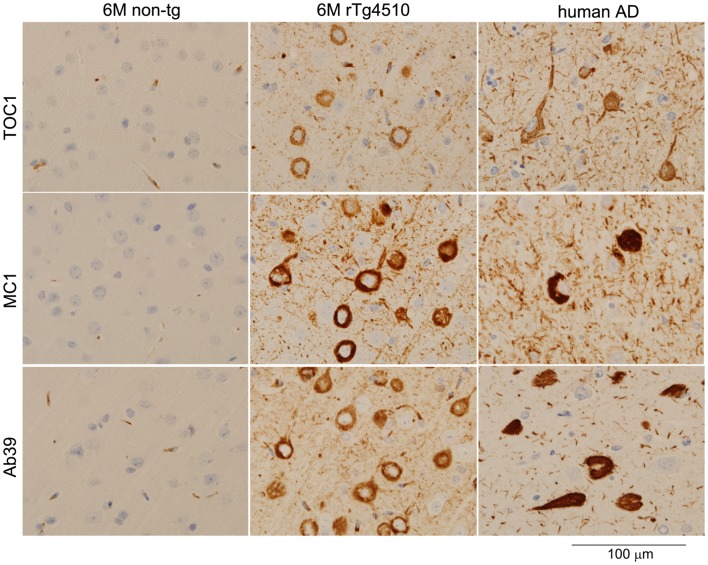
**Light microscopic images of immunostained brain sections from a non-transgenic mouse, a rTg4510 mouse, and human with AD**. Formalin-fixed paraffin sections were stained with TOC1 (1:2500), MC1 (1:1000), and Ab39 (1:250) antibodies by the Dako Universal Autostainer (Dako, Carpinteria, CA, USA). The sections were then counterstained with hematoxylin. TOC1 diffusely stained cytoplasmic regions of neurons (top panels, rTg4510 and AD brains), while MC1 and Ab39 densely stained these neurons (middle and bottom panels, respectively). This difference in staining pattern is due to the specific binding of TOC1 antibody to premature tau aggregates. Scale Bar, 100 μm.

TOC1 immunoreactivity is selectively detectable in the early stage of AD pathogenesis; however, TOC1 antibody fails to label mature tangles in AD brains (26). In rTg4510 mice, TOC1 immunoreactivity was observed in the TBS-extractable 64 kDa tau enriched fraction and linked to early pathological changes (Sahara, in preparation). Notably, the immunohistochemical staining pattern of TOC1 antibody was clearly different from those of MC1 and Ab39 antibodies (Figure 1). Since NFTs themselves might be protective [reviewed in Ref. (41)], other harmful tau species such as tau oligomers are currently of particular interest. If TOC1 antibody selectively interacts with tau dimers and higher-order oligomers but not tau filaments, and if those species cause neurotoxicity, this antibody can be a useful tool to track the pathway of tau neurotoxicity.

Another tau oligomer-specific antibody, T22, was generated by Kayed’s group (42) against antigenic tau prepared from Aβ seeding of tau oligomers (35). The specificity of this antibody was confirmed by ELISA and dot blotting. It detects only tau oligomers but not tau monomers or PHF fibrils prepared by the heparin-induced tau polymerization method (42). Immunohistochemically, this antibody selectively stains pretangles, neuritic plaques, and neuropil threads but not ghost tangles in AD brain sections (42). Western blotting showed that T22 antibody recognizes higher-molecular-weight tau species (e.g., dimers, trimers, and tetramers) but not monomers (42). It should be noted that the SDS-PAGE samples were not denatured by boiling before running on gels and that T22 immunoreactivity was diminished by denaturing agents such as 8 M urea (42).

These novel tau oligomer-specific antibodies provide a new method to diagnose the early pathological changes that occur in tauopathy. It would be extremely useful to develop methods employing cerebrospinal fluid biomarkers combined with total tau, phosphorylated tau, tau oligomers, and other biomarker measurements to differentially diagnose dementias, such as AD, frontotemporal lobar degeneration, progressive supranuclear palsy, corticobasal degeneration, dementia with Lewy bodies, vascular dementia, and prion disease.

## Conclusion

Abnormal tau aggregation is considered to be a critical pathological feature of tauopathy. However, the initial molecular event of tau pathogenesis is yet unclear. The hyperphosphorylation of tau is strongly suggested to be directly correlated with the severity of AD pathology (43). Iqbal and colleagues demonstrated that hyperphosphorylated tau extracted from AD brain reduces MT stabilization, sequesters normal tau from MT, and aggregates themselves in the absence of inducer molecules (20, 44). Many studies attempting to identify tau oligomers have demonstrated the existence of hyperphosphorylated tau oligomers in human and transgenic mouse brains (e.g., AD P-tau, 140 and 170 kDa tau multimers, TBS-extractable 64 kDa tau, and T22 antibody-positive tau oligomers) (Table 1). Thus, hyperphosphorylation of tau could be the initial event of NFT formation. However, the amount of 140 kDa tau multimer (normal tau dimer) correlates well with behavioral deficits (14), suggesting that hyperphosphorylated tau oligomers may not be essential for neurotoxicity. If antibodies can be generated to recognize non-phosphorylated and hyperphosphorylated tau dimers independently, we will be able to better identify toxic tau species and optimize potential oligomerization inhibitors as possible novel therapies. Standardized isolation methods of tau oligomers are in need to improve consistency between researchers.

**Table 1:** **Summary of brain-derived tau oligomer preparation methods**.

Product	Reference	Material origin	Detect method	Oligomer properties
AD P-tau	Kopke et al. (12), Alonso et al. (20), Alonso et al. (44)	AD patients	WB, EM	AD P-tau is isolated from the 27 K–200 K × *g* fraction, soluble in urea, hyperphosphorylated, no ubiquitin immunoreactivity, self-assembly into filaments, sequesters N-tau
140 and 170 kDa tau	Berger et al. (14)	rTg4510 mice JNPL3 mice	WB, SEC	Disulfide-bond independent, correlates with memory loss, 140 kDa tau is not hyperphosphorylated, 170 kDa tau is hyperphosphorylated, and has strong immunoreactivity with AT8
TBS-extractable 64 kDa tau	Sahara et al. (25)	rTg4510 mice	WB, IHC, EM	TBS-extractable 64 kDa tau is isolated from 27 K to 150 K × *g* fraction, thermo-stable, hyperphosphorylated, mostly disulfide-bond-dependent, correlates with brain atrophy, contains tau-positive granules/short filaments
T22-positive tau	Lasagna-Reeves et al. (38), Lasagna-Reeves et al. (42)	AD patients	WB, IHC, SEC, AFM	Hyperphosphorylated, not ubiquitinated at pretangle stage, contains oligomers with 4–8 nm diameters, propagate abnormal tau conformation of endogenous murine tau

*WB, Western blot; EM, electronic microscopy; IHC, immunohistochemistry; SEC, size-exclusion chromatography; AFM, atomic force microscopy*.

In summary, accumulating evidence from biochemistry, immunology, and molecular imaging reveal the existence of tau oligomers as mainly buffer-soluble, non-filamentous, granular-shaped conformers. The neurotoxicity of these oligomers has been confirmed in both *in vitro* and *in vivo* experiments (4, 5, 14, 42, 45). The next step of tau oligomer research should investigate whether tau dimers and/or non-granular oligomers exist and functionally correspond to neuronal dysfunction.

## Conflict of Interest Statement

The authors declare that the research was conducted in the absence of any commercial or financial relationships that could be construed as a potential conflict of interest.
